# Online Chats to Assess Stakeholder Perceptions of Meat Chicken Intensification and Welfare

**DOI:** 10.3390/ani6110067

**Published:** 2016-10-27

**Authors:** Tiffani J. Howell, Vanessa I. Rohlf, Grahame J. Coleman, Jean-Loup Rault

**Affiliations:** Animal Welfare Science Centre, Faculty of Veterinary and Agricultural Sciences, The University of Melbourne, Alice Hoy Building (162), Parkville, VIC 3010, Australia; tiffani.howell@unimelb.edu.au (T.J.H.); vanessa.rohlf@unimelb.edu.au (V.I.R.); Grahame.Coleman@unimelb.edu.au (G.J.C.)

**Keywords:** forums, meat chickens, broilers, intensive farming, free-range, natural behaviour, livestock, animal welfare

## Abstract

**Simple Summary:**

Most people care about animal welfare. Nevertheless, divergent views remain on what constitutes animal welfare, despite a growing body of scientific evidence. We used online chats to trigger discussion among participants from various stakeholder groups: general public, animal advocacy group, meat chicken industry-affiliated, and researchers or veterinarians who were not industry-affiliated but had experience with chickens. The aim of this pilot study was to assess reasons for divergence in opinions or conversely agreement between participants, using the topic of the welfare implications of meat chicken farming intensification. Participants also completed a pre- and post-chat survey to evaluate their perceptions and knowledge of chicken farming. Reasons for supporting intensification included perceptions of better health for the chickens and the sustainability of the system. Reasons for opposition included perceptions of the large number of animals kept together, and limited ability to perform natural behaviours. Misunderstandings about current practices were clarified in chats which contained industry-affiliated participants. Participants agreed on the need for enforceable standards and industry transparency. On average, objective knowledge of intensification increased after participating in the chat, but support for intensification did not change over the course of the study, counter to assertions that lack of knowledge results in lack of support for some practices. Engaging stakeholders can provide valuable information to anyone interested in the relationship between perception and knowledge of specific farming practices.

**Abstract:**

Evidence suggests that there is variation in support for specific chicken farming practices amongst stakeholder groups, and this should be explored in more detail to understand the nature of these differences and work towards convergence. Online focus groups were used to assess attitudes to animal welfare in meat chicken farming in this pilot study. Across six online chats, 25 participants (general public, *n* = 8; animal advocacy group, *n* = 11, meat chicken industry, *n* = 3; research or veterinary practice who had experience with poultry but no declared industry affiliation, *n* = 3) discussed meat chicken intensification and welfare. Of those, 21 participants completed pre- and post-chat surveys gauging perceptions and objective knowledge about meat chicken management. Main reasons for intensification support were perceptions of improved bird health, and perceptions that it is a cost-effective, sustainable farming system. Reasons for opposition included perceptions that a large number of birds kept are in close proximity and have limited ability to perform natural behaviours. Misunderstandings about current practices were clarified in chats which contained industry representation. Participants agreed on the need for enforceable standards and industry transparency. Industry-affiliated members rated welfare of meat chickens higher, and gave lower ratings for the importance of natural living, than other stakeholder groups (both *p* = 0.001). On average, while objective knowledge of intensification increased after chat participation (*p* = 0.03), general welfare ratings and support for intensification did not change over time, counter to assertions that lack of knowledge results in lack of support for some practices.

## 1. Introduction

Animal welfare is an increasingly important societal consideration across many industrialised nations [1,2,3,4]. Central to this increased concern for animal welfare are questions surrounding the treatment and care of livestock animals. Consistent with this, there is increasing demand for animal products perceived to be “welfare-friendly”, including organic and free-range foods [5,6].

Despite this growing interest in animal welfare, there remains much difference of opinion among stakeholders [7,8,9,10], including disagreements over aspects as fundamental as the definition of and means to assess animal welfare [10,11,12,13]. The general public, including consumers of animal products, emphasise the presence or absence of positive mental states (i.e., pleasure) and management styles associated with “natural living” when defining livestock animal welfare [4,10,13,14]. Adequate space [15], freedom to roam [10,15,16], social contact with other animals [10], and freedom to fulfil natural desires [16] are commonly cited by the public as indicators of good livestock animal welfare.

Industry representatives, on the other hand, typically consider livestock animal welfare in biological functioning terms [12,13,16]. Physical health, fertility, and other aspects associated with production are barometers of livestock animal welfare for many industry members [12,13,17]. These different conceptions of what constitutes animal welfare can lead to certain farming practices and housing systems being favoured over others. Those who consider animal welfare in terms of biological functioning are more likely to prefer the provision of indoor housing, where the environment and animals can be regulated and closely monitored, and which are typically more cost-effective, over other housing conditions [17,18]. In contrast, those who value natural living are more likely to favour outdoor access and free-range environments [10,19].

These somewhat opposing philosophies can hinder progress in the field of animal welfare. As with any conflict, the best means of resolution is to identify and understand stakeholder concerns [20]. It is only by identifying areas of common ground and areas of dissent that progress can be made and resolutions found [20].

While there is empirical knowledge regarding stakeholder conceptions of livestock welfare in general [19,21], relatively little is known about stakeholder opinions toward industry-specific farming practices. Exceptions to this are recent studies exploring stakeholder opinions towards farming practices in the dairy [22,23,24] and pork [14] industries. However, studies identifying stakeholder opinions towards practices in the chicken meat industry are limited. A Belgian study comparing consumers and producers of meat chickens found that both groups place high importance on meat chicken welfare [25]. However, in the same study, consumers disagreed with producers that people would be unwilling to pay more for high welfare products, or that meat chickens suffer little [25]. A Dutch study found that outdoor access is the most important attribute in consumer perceptions of meat chicken welfare, followed by stocking density [26].

Further research is required to better understand whether specific practices, such as intensification, in meat chicken management are perceived to impact on bird welfare. Additionally, the perceptions of animal welfare for meat chickens raised in intensive systems should be considered alongside the relative environmental sustainability of this management system compared to other farming systems. Intensively farmed chicken meat is one of the cheapest and most environmentally sustainable sources of animal protein, based on a life-cycle analysis of land use and carbon footprint [27]. However, the United Nations Food and Agriculture Organization considers animal welfare to be an indicator of sustainability [28]. It is unclear whether the general public believes that improved production outcomes are important enough to justify any perceived negative impacts on the welfare of individual animals, such as lack of opportunity to engage in some natural behaviours. Identifying the beliefs underlying stakeholder decisions to oppose or support particular farming practices will provide information to direct outreach or research strategies that bring industry, the public and other stakeholders’ perceptions into further alignment.

In order to accomplish this, it is necessary to explore how and why opinions change in response to the provision of information. It is sometimes argued that one major factor underlying stakeholder differences of opinion is differences in levels of knowledge. Specifically, some industry members argue that public opposition towards certain farming practices is the result of lack of knowledge [14]. Indeed, knowledge is an important antecedent of beliefs [29], and research indicates that the public has only a vague understanding of animal welfare issues, at least in Western societies [1,16,17]. Studies investigating stakeholder beliefs underpinning levels of support for or opposition to specific farming practices should therefore also measure levels of industry and farming practice knowledge held by the stakeholder groups. This information will be useful to determine whether supporting or opposing specific farming practices may be the result of lack of knowledge or misinformation about a particular issue, thus highlighting areas where communication is necessary. For instance, providing information to consumers about the different types of housing systems available for pregnant pigs or meat chickens result in decreased support [14,30]. In this case, the provision of information did modify the opinions of some members of the public, although not by increasing support, as some industry members may have expected.

Previous studies have used a variety of methods to probe stakeholder opinions, including interviews [4,15], surveys [3,8,31], and face-to-face focus groups [7]. Focus groups are a useful means of gathering information when few empirical data exist on the topic [32]. Conventional focus groups are face-to-face discussions, and while they are useful in providing a rich understanding of a particular topic, they do have a number of limitations, such as constraints on time and distance. For this reason, online discussions have recently been employed in Canada as a successful and low-cost means to explore animal welfare views [14,22,33]. An additional advantage of these virtual focus groups is that participants’ identity can be protected. This can be useful in encouraging honest feedback and stimulating group discussions about topics that may be controversial [34]; “online discussion provides a safe and productive format for discussions about contentious issues …” ([22], p. 3831). The aim of this pilot study was to engage and explore stakeholder perceptions and objective knowledge of meat chicken intensification and welfare using surveys and online chats.

## 2. Materials and Methods

This study received ethics approval from the University of Melbourne Human Research Ethics committee (approval number 1545125.1).

### 2.1. Participants

All participants were required to be at least 18 years of age, resident in Australia, and able to read and write in English. A total of 25 participants took part in the online chats, and 21 participants completed all components of the study. Of these 21 participants, three identified as meat chicken industry-affiliated, eight as animal advocacy group members, and eight as general public. The final two participants were in research (*n* = 1) or veterinary practice (*n* = 1), but with no declared industry affiliation or animal advocacy group affiliation. These two participants were categorised separately based on free-text responses provided when asked which group they identified with most. These responses indicated a level of experience with chickens that may have exceeded that of many members of the general public, but they had not declared any experience with the meat chicken industry. Participants were 67% female (*n* = 14), and mean age was 42 years (SD = 15 years).

### 2.2. Materials

A windows-based content management system (AWSC-CMS) was created specifically for this project. This system can be used on most internet browsers (e.g., Mozilla Firefox, Internet Explorer, Google Chrome and Safari), and on multiple platforms including Windows, Mac and iPhones. With a customisable Home page, the content management system allows the user to create and administer multiple surveys, as well as conduct traditional forum discussions and online live chats.

Pre- and post-chat surveys were developed, which were each expected to take approximately 10–15 min to complete. The pre-chat survey consisted of 12 items divided into three sections. Section 1 focused on participant demographics and which stakeholder group they primarily identified with. There were also two items asking participants to rate their own knowledge of the Australian chicken meat industry, and chicken husbandry and welfare, with response options for both items ranging from 1 (very low) to 5 (very high). Section 2 contained questions relating to objective knowledge of chicken management practices in the meat chicken and laying hen industries (see Table 1). The item related to beak trimming was included even though beak trimming is not practiced in meat chicken management, because we simultaneously ran a study on perceptions of laying hen welfare and management, and we intended to compare the responses of participants in the current study to the responses of participants in the laying hen study. Results of the laying hen management study will be presented in a future report.

Section 3 contained items related to beliefs about chicken welfare, farming, and housing (adapted from [35]). We asked participants to rate the importance of a variety of factors for chicken welfare, with response options ranging from 1 (not at all important) to 5 (very important). These factors were: outdoor access, protection from extreme weather, social contact with other chickens, ability to engage in natural behaviour, protection from predators, preventive medicine (e.g., antibiotics), medicine to treat disease, and protection from aggression from other chickens. Participants also rated the welfare of meat chickens farmed in intensive farming conditions and in free-range farming systems, with response options ranging from 1 (very low) to 5 (very high). A “don’t know” option was provided for this question. We asked participants to rate their level of support for intensive farming practices in the chicken meat industry, with possible response options ranging from 1 (strongly oppose) to 5 (strongly support); participants could also select “don’t know”. All “don’t know” responses were coded as 3 (mid-point) for analysis.

The final questions asked participants whether they had accessed outside information when completing the survey, their general availability to attend a chat, and where they heard about the project. The post-chat survey contained the nine questions presented in Section 2 and Section 3 of the pre-chat survey, as well as a free-text item asking participants to explain why they supported or opposed intensification.

### 2.3. Procedure

Recruitment advertisements were sent to contacts in the meat chicken industry, animal advocacy organisations, and social media (e.g., Facebook, the University of Melbourne staff newsletter). Interested, eligible participants were asked to contact the research team by email, who registered them on the AWSC-CMS system and gave them access to the pre-chat survey.

After participants had completed the pre-chat survey, the research team organised a series of six different chats across three weeks. Between five and seven people were invited to each chat, which consisted of either a single stakeholder group (e.g., general public only), or more than one group (e.g., general public, animal advocacy, and/or industry-affiliated). Three of the chats were attended by all of the participants who were invited to that particular chat, including both mixed-group chats with representation from industry, and the chat with only animal advocacy group members. There were two mixed group chats with no industry representation, and of those, two participants attended one, and three participants attended the other. The sixth chat, which consisted only of members of the general public, had two participants. All chats were moderated by one of the authors (Tiffani Howell).

Each chat lasted between 60 and 90 min. An outline of the chat, including a scientific statement explaining meat chicken intensification, was provided to the participants during the chat (Table 2). The statement was written based on scientific evidence, and refined in consultation with industry and an animal advocacy group. The goal was a statement that could be easily understood by members of the lay community, with an objective summary of the advantages and disadvantages of intensification for meat chicken farming. Two days after the chat, participants were emailed an invitation to complete the post-chat survey.

### 2.4. Analysis

Descriptive frequency statistics permitted the observation of trends in the data. We used Fisher’s exact tests to measure differences between groups on objective knowledge of chicken industry practices, as these data were dichotomous (correct/incorrect). We used McNemar’s tests to measure differences on objective knowledge over time.

In order to reduce the number of variables included in analysis, three composite variables were created. One composite variable included three items measuring welfare ratings of chickens in intensive and free-range systems, and support for intensification (Cronbach’s alpha = 0.82). This composite variable was named “general welfare”. The second composite variable, called “natural living”, included three items measuring the perceived importance of social contact with other chickens, outdoor access, and the ability to engage in natural behaviours (Cronbach’s alpha = 0.76). The third composite variable, called “protection”, included five items measuring the perceived importance of protection from extreme weather, protection from predators, protection from aggression from other chickens, the use of preventive medicine, and the use of medicine to treat disease (Cronbach’s alpha = 0.88).

A mixed model ANOVA was used to measure differences between different stakeholder groups across time for the three composite scores as well as the items measuring perceived knowledge of chicken husbandry and welfare, and the Australian meat chicken industry. Data were normally distributed for these five items. Group differences are reported as F-values, and Wilk’s λ was used to report differences across time. For Fisher’s exact tests, McNemar’s tests, and ANOVAs, we included the following groups: general public (*n* = 8), animal advocacy group members (*n* = 8), and industry-associated participants (*n* = 3). Because two participants did not fit into any of these groups, they were excluded from this analysis, which therefore included a total of 19 participants. Due to the low representation in some groups, results from the present pilot study should be interpreted with caution.

Chat transcripts were analysed qualitatively for recurring themes. NVivo analysis software (QSR International, Doncaster, Victoria, Australia) and Tagul online software (www.tagul.com) were used to generate word clouds of the 75 most commonly used words in the chats. For the purposes of the word cloud, we excluded words with less than 4 letters (e.g., “the”, “and”, “a”), and conversational fillers such as “think”, “believe”, “thanks”, and “would”. This qualitative analysis included text data from 25 participants in the chats.

## 3. Results

A total of 25 participants took part in six chat discussions. Group composition, number of posts, and number of words for each chat is reported in Table 3.

Four participants did not complete the post-chat survey even after being sent email reminders to do so. Therefore, we have included pre- and post-chat data from two participants in Chats 1 and 5, and all participants from all other chats, for a total of 21 participants.

### 3.1. Qualitative Analysis of In-Chat Texts

#### 3.1.1. Stakeholder Group Differences in Meat Chicken Intensification Perceptions

Perhaps unsurprisingly, industry-affiliated participants during the chats appeared more likely to support meat chicken intensification than members of animal advocacy groups or the general public. Among participants who indicated that they did support intensification, some of the reasons for support included perceptions of improved health for the birds and the perception that it was a cost-effective, environmentally-sustainable farming system which provides low-cost protein to a growing population.

“*…if chickens were not grown as efficiently as they are, we would have a lot more people struggling to feed their families, and the Australian economy would look pretty sick.*”

Among participants who indicated that they did not support intensification, reasons for opposition included perceptions of the large number of birds kept in close proximity to one another, and perceived limited ability to engage in natural behaviours.

“*Chickens raised in any sort of intensive operation are not able to perform the behaviours that are characteristic of their species, such as dust-bathing and scratching for food. Nor are most chickens able to enjoy fresh air and sunshine.*”

#### 3.1.2. Clarifying Meat Chicken Management Practices

There was a fair degree of misunderstanding about specific chicken management practices among members of the general public and animal advocacy groups. For instance, several participants indicated that they disagree with the use of hormones and beak trimming in meat chicken farming.

“*(Welfare concerns include) de-beaking without anaesthetic.*”

“*...They are injected with steroids to make them grow faster.*”

In the two chats where industry-affiliated participants interacted with members of animal advocacy groups and the general public, this provided an opportunity to clarify that beak trimming is not practiced, and that hormones have not been used in decades. In the case of beak trimming, this confusion may have been due to the pre-chat survey item related to beak trimming, which may have caused some participants to believe that it occurred in the meat chicken industry.

“*There is no de-beaking in meat birds practiced.*”

“*There are no hormones in chicken feed here in Australia, and there has not been for approximately 50 years.*”

#### 3.1.3. Common Ground between Stakeholder Groups

While there was considerable disagreement about the way chickens should be managed, there was agreement between members of different stakeholder groups on two key topics: enforceable standards and transparency within industry.

“*CCTV (closed-circuit television) footage live streamed in all CAFOs (concentrated animal feeding operations)…might address accountability.*”

“*I would like to see certain standards implemented in a law and communicated transparently. We all should know what stocking density to deal with, what welfare parameters have to be considered, etc. Currently, there is too much confusion, misleading information, and different accreditation bodies.*”

#### 3.1.4. Relative Importance of Environmental Sustainability and Animal Welfare

We expected that concerns about animal welfare in intensive systems would be more important to participants than any potential environmental benefits, among participants who did not support intensification. The scientific statement introduced during the chat mentioned that intensive meat chicken is a relatively environmentally sustainable way to produce animal protein. Some chat participants did appear concerned about the sustainability of livestock farming; however, participants who did not support intensification doubted the sustainability of producing any type of meat, including chicken meat. Some of these participants suggested that plant-based food sources should be used more often than meat, as humans do not need meat to survive.

“*If we would all just calm down and not demand meat with every meal there would be no need for intense farming.*”

“*Yeah I seriously doubt the sustainability of chicken meat farming, at least compared to crops.*”

Other participants indicated the low likelihood of most consumers becoming vegetarian or vegan in the near future, and acknowledged the complexity of the issue.

“*I think it is a lovely, but unrealistic, dream for people to eat less meat or no meat at all. People don’t care enough.*”

“*It certainly is an intricate puzzle.*”

### 3.2. Comparing Pre- and Post-Chat Survey Results

On average, participants reported a moderate level of knowledge about Australian meat chicken practices, and chicken welfare and husbandry, before the chat. Full descriptive results of pre- and post-chat ratings by group, and effects of time and group, are available as Supplementary Materials. We analysed whether there were any differences across time and between stakeholder groups. For both of these items, there were changes across time, and differences between groups. For the item related to chicken husbandry and welfare knowledge, there was a significant increase in self-rated knowledge after chat participation among all groups (Wilk’s λ = 0.77, *F* (1,16) = 4.69, *p* < 0.05, partial eta squared = 0.23). Also, industry-affiliated participants reported a higher level of knowledge than other groups (*F* (1,28) = 9.69, *p* < 0.01, partial eta squared = 0.55). The item investigating Australian meat chicken industry knowledge similarly showed a significant increase in self-rated knowledge after chat participation among all groups (Wilk’s λ = 0.70, *F* (1,15) = 6.58, *p* = 0.02, partial eta squared = 0.31), and industry-affiliated participants demonstrated a higher level of knowledge than other groups (*F* (2,15) = 7.51, *p* < 0.01, partial eta squared = 0.50).

#### 3.2.1. Objective Knowledge of Meat Chicken Farming Practices

The percentage of correct responses for the items probing objective knowledge of chicken management practices was variable (Table 4), ranging from 43% for intensification, to 95% for beak trimming.

There were no significant differences between stakeholder groups in the percentage of correct responses for these items (Fisher’s exact tests). However, there was a difference observed across time for the item related to intensive meat chicken farming (McNemar’s test, *p* = 0.03). More participants answered correctly after participating in the chats compared to the pre-chat survey.

#### 3.2.2. Factors Affecting Welfare and Perceived General Welfare of Meat Chickens

Two composite scores, “natural living” and “protection”, were generated from the eight items related to perceived importance of various factors for meat chicken welfare. For full descriptive results, please see the Supplementary Materials.

There was no effect of time on either of the two composite scores, but there was a significant difference between groups on the “natural living” score (*F* (2,16) = 10.09, *p* = 0.001, partial eta squared = 0.56). Animal advocacy group members and members of the general public placed higher importance on aspects of natural living than industry-affiliated participants.

A third composite score, called “general welfare”, was created from the item measuring support for intensification and items measuring perceptions of chicken welfare in free-range systems and intensive systems. For full descriptive results, see the Supplementary Materials. There was no effect of time on this score, but there was a difference between groups (*F* (2,16) = 12.52, *p* = 0.001, partial eta squared = 0.61). Industry-affiliated participants rated the welfare of meat chickens higher than animal advocacy group members or members of the general public.

#### 3.2.3. Reasons for Support for or Opposition to Meat Chicken Intensification

All participants were invited to provide free-text responses explaining their support for or opposition to meat chicken intensification, based on their experience in the chat. Participants who expressed support for intensification described it as an efficient way to feed protein to a growing population:

“*As a farmer of 30 years’ experience I have seen huge advancement of shed technology which allows the industry to be a very efficient producer of protein, while providing a high level of animal welfare to the chickens in our care.*”

Another participant highlighted the relative infrequency of disease and food safety risks in intensive systems:

“*This is the safest way to grow chickens and minimise disease and food safety risks, and at the end of the day we are growing the birds to provide food.*”

Most chat participants did not support intensification of meat chickens, overwhelmingly due to concerns for animal welfare. Some participants expressed concerns that the chickens were unable to exhibit natural behaviours:

“*This practice does not allow chickens to perform natural behaviours and interact socially and naturally with other chickens.*”

Some individuals disapproved of current practices in meat chicken management:

“*Through selective breeding, chickens in intensive situations also grow abnormally large, becoming top heavy, and causing damage to their legs. They are also killed while they are effectively babies.*”

Some participants indicated concern about lack of transparency in the industry:

“*It is very difficult to allocate a degree of support for intensification practices when you have insufficient information to judge. My perception is that this is the challenge for many Australians. We lack a clear and simple set of standards that are immediately transparent to the ordinary consumer in the market. Many citizens would like to know what effects intensification has on the comfort and health of the chickens during their short lives. Having such information immediately available would enable an informed decision of conscience.*”

Finally, several participants who opposed intensification viewed the management system as a triumph of greed over ethics:

“*Sometimes there needs to be a balance between the welfare of lives and profit, and in meat chicken rearing systems the focus is only on profit.*”

#### 3.2.4. Access to External Sources of Information

Seven participants indicated that they accessed external sources to help them during the course of the project, including four who accessed information before the chat, six of whom accessed info during the chat, and one who accessed it while filling out the post-chat survey. Of these, animal advocacy groups Animals Australia and the Royal Society for the Prevention of Cruelty to Animals (RSPCA) were common sources of information. One participant also reported looking up the Animal Welfare Science Centre’s associations and funding bodies.

## 4. Discussion

Online surveys and chats were an effective way of gathering information about perceptions and reasons for approval or disapproval of the intensification of meat chicken farming practices, as well as objective knowledge of poultry farming practices. In two of our chats, industry-affiliated participants interacted with members of the general public and animal advocacy groups. Self-assessed knowledge of the Australian meat chicken industry, and of chicken husbandry and welfare, increased after participating in the chats. Objective knowledge about meat chicken intensification also increased. As a rule, participants rated the general welfare of meat chickens quite low, but industry-affiliated participants rated it much higher than participants in the general public or animal advocacy groups. These ratings did not change significantly over the course of the study. This is counter to arguments that lack of understanding about industry practices causes lack of support from the general public [14].

It is important to understand beliefs towards specific animal welfare issues, but it is also important to examine why and how opinions may change based on new information. When U.S. and Canadian participants were presented with information about housing systems for pregnant pigs, support for gestation stalls decreased from 30.4% to 17.8% [14]. Because there were multiple sources of information, and access to the information was voluntary, it is not clear which aspects of the information presented were responsible for the opinion change.

In the current study, all participants were provided the same information, by way of the scientific statement. Support for intensification did not increase after objective information was provided. However, it was impossible to control whether people accessed additional information from other sources. Indeed, seven participants indicated that they did seek information elsewhere during the study, which was predominately sourced from animal advocacy group websites rather than industry or scientific reports.

Participants who supported intensification cited the cost-effective nature of this farming method, as well as the relative environmental sustainability of intensive chicken systems compared to other livestock farming methods. In response to the statement that intensification is environmentally friendlier than other systems, some participants who opposed intensification suggested that meat should just be eaten less than it currently is. However, other participants doubted that this profound cultural shift is likely to occur in the short to medium term.

A key practical implication of this work is the role that consumers play in determining industry practices. Generally, the public believe animal welfare is important [1,2,3,4]. However, animal welfare knowledge is generally gained passively from word of mouth, documentaries or news media [16]. Of the public that do actively seek information, the most common sources of information are animal advocacy groups such as the RSPCA (Royal Society for the Prevention of Cruelty to Animals) and HSUS (Humane Society of the United States), and it is rarely sought from sources like agricultural industry websites [1,3]. It is not currently known what levels of knowledge individual animal advocates have towards specific animal welfare issues. One recent study found that opinion leaders, who are more likely to be members of animal advocacy groups, have the same levels of actual knowledge as the general public, but their self-assessed level of knowledge is higher [1]. This was not the case for the current study, in which perceived knowledge was higher for members of the industry than for the general public or animal advocacy group members. However, there was no significant difference between stakeholder groups on objective knowledge; correct responses in the pre-chat survey varied between 40% and 70% of all participants, with the exception of beak trimming, the correct description of which was correctly identified by nearly all participants in the pre-chat survey.

The inclusion of an item related to beak trimming on the surveys may have confused participants into thinking that it was practiced for meat chicken management. This item was included because of a similar study we ran simultaneously related to laying hen management, and we planned to compare objective knowledge between participants in both studies. Participants might not have thought that beak trimming occurred in meat chickens if they had not been exposed to this item in the pre-chat survey. Indeed, objective knowledge of beak trimming actually decreased over the course of the study, although this result was not significant.

There could also have been confusion about the items related to use of antibiotics and intensive meat chicken farming. It is possible that some farmers use antibiotics to increase chicken growth rate, but this was deemed an incorrect response for the current survey as the main intent in Australia is to prevent and treat diseases. Similarly, for the item related to intensification, some countries house meat chickens in cages. We only recruited Australian participants, and the recruitment ad and plain language statement both mentioned that we would be discussing Australian meat chicken practices. However, if participants were aware of practices overseas, they may have mistakenly believed that these practices also occurred in Australia.

It is possible that approval of practices is related to the importance that people place on certain attributes to the wellbeing of livestock animals. In one study, most people rated husbandry attributes, such as good ventilation and nutrition, as more important than natural living attributes, such as freedom to roam outdoors and contact with offspring [35]. However, the more important people rated natural living attributes, the less likely they were to approve of livestock practices.

For meat chickens, intensification was perceived by many participants in this study as forcing animals to live in an unnatural way, with little opportunity to engage in natural behaviours, and in groups far larger than they should experience. The scientific statement did not dispel any of these concerns, instead focusing on the relative environmental sustainability and improved productivity to feed a growing population. This could explain why the welfare ratings for chickens did not change after participating in the chat. Future research should examine whether objective information about improvements to natural living attributes for specific livestock management practices results in perceptions of higher welfare for those animals.

Some limitations to this study should be noted. For instance, responses to the survey items related to perceived importance of different factors which could impact chicken welfare were biased toward high importance ratings. Future research should aim to develop survey items that do not encourage this type of bias, as ceiling effects may have negatively impacted the ability to draw comparisons between different participants.

The scientific statement provided in the chat was intended to convey as much information as possible, as concisely as possible. This meant that some nuance was inevitably lost, and perhaps a more accurate picture of the benefits and draw-backs of intensification could have been provided in a longer piece. We made it brief so that participants could quickly read and make sense of the information provided in the statement and respond accordingly. We think it is unlikely that a more in-depth explanation of intensification would have changed the results.

A comparison between meat chickens in free-range systems and intensive farming conditions is a somewhat false comparison, as it could be argued that some chickens in free-range systems are also kept intensively. In hindsight, we should have specified “intensive indoor systems” rather than “intensive farming conditions”, since some free-range systems could be considered intensive. This was correctly stated in the scientific statement, but not the survey question. It is likely that most people consider intensive systems to be indoors, but future research should clearly differentiate these types of systems. Additionally, we did not obtain information from industry-affiliated participants about their specific role within the industry. Future research should aim to understand whether industry-affiliated participants vary depending on their industry role or experience, as they are not necessarily a homogenous group.

This project included a small, convenience sample of participants. It uncovered a broad range of beliefs underlying decisions to approve or disapprove of livestock practices. However, the findings contained in this report are not statistically representative and results cannot be generalised to the public, industry or animal welfare groups. In particular, animal advocacy group members were over-represented in relation to their proportion in the wider community. Furthermore, we did not ask participants whether they were vegetarian, vegan, or meat-eaters, which may also bias the outcomes. The dynamic of the conversations may be considerably different if future research uses a more representative sample of participants. Additionally, participants completed the post-chat survey within a few days of participating in the chat discussions. It is possible that opinions or knowledge levels were changed on the short-term, but whether this has medium- or long-term impacts is unclear based on the current study. We recommend that future research of this kind should incorporate a larger number of participants over a longer time span.

## 5. Conclusions

In this pilot study with 25 participants, attitudes toward meat chicken intensification varied between stakeholder groups, with industry-affiliated stakeholders more supportive of intensification than the general public or animal advocacy group members. Online surveys and chats were an effective way of gathering information about perceptions and reasons for approval or disapproval of livestock farming practices, as well as objective knowledge of those practices. Future research should examine whether other farming practices differ in terms of stakeholder perceptions, particularly practices that are perceived to increase the opportunity for animals to engage in natural behaviours.

## Figures and Tables

**Table 1:** Items measuring objective knowledge of chicken management practices.

Item	Response Options
Free range chickens are chickens that:	Have the ability to access an outdoor area as they please, at least during the day **^1^** Are free to roam around in a large shed Have access to an outdoor area for their entire lives, from the day they hatch Have access to the outdoors and are bred and reared without the use of chemicals and hormones
Beak trimming:	Involves the removal of a chicken’s entire beak to prevent disease Involves the removal of the chicken’s entire beak to prevent feather pecking and cannibalism Involves the removal of the tip of the chicken’s beak to prevent feather pecking and cannibalism **^1^** Involves the removal of the tip of the chicken’s beak to prevent disease
Antibiotics:	Are administered to chickens to prevent and treat infections and disease **^1^** Are administered to individual chickens showing symptoms of illness, to treat infections and disease Are not administered to chickens in Australia Are administered to increase the rate of growth in chickens
Intensive meat chicken farming involves:	Keeping meat chickens indoors in high densities **^1^** Feeding growth hormones to chickens to increase their rate of growth and size Housing meat chickens in cages All of the above
Intensively raised meat chickens are typically slaughtered at:	Six to 12 months of age Two years of age 5 to 7 weeks of age **^1^** 12 weeks of age

**^1^** Correct response.

**Table 2:** Chat discussion plan.

Chat Segment	Text (Presented by Moderator)
Welcome statement	Hi everyone. Welcome to the discussion forum. Thanks again for your time today. Just to remind you all that the aim of this discussion is to discuss your thoughts on meat chicken welfare and intensification in Australia. The estimated time for this part of the research project is approximately 1–2 h.
First post	I’d like to begin the discussion by asking you all what your general thoughts are on meat chicken welfare in Australia. Generally speaking, do you think meat chickens have good or bad welfare?
*(follow chat and ask probing questions as necessary)*	
Intensification introduction	One type of farming practice for meat chickens in Australia is intensification. Have you heard of it before? If so, what do you think about it?
*(follow chat and ask probing questions as necessary)*	
Introduce intensification scientific statement	Intensification can be defined as changes towards more confined production systems and the concentration of production on fewer farms, aimed at increasing the efficiency of production. It does not necessarily mean indoor housing, as some systems which provide access to an outdoor area can also be considered intensive. Chicken meat is the cheapest source of animal protein, with the smallest carbon footprint for animal production, making it an environmentally and economically sustainable production system to feed a growing human population. Many of these goals have been achieved through intensification. Intensification of meat chicken production involves improved chicken nutrition and health management for the flock as a whole, larger flocks (30,000+) of meat chickens kept at higher densities, and chickens which are bred to grow bigger and faster through genetic selection. However, some individual chickens have anatomical (for example, lameness) and metabolic (for example, heart disease) problems, mostly resulting from intense genetic selection for rapid growth. Chickens live in large groups with limited choice of living environment or pen mates. Often, chickens have behavioural restrictions, such as limited space to move around at the later stage of the growing period. They also often have less environmental stimulation, such as limited opportunity to forage and explore. Do you oppose or support this method of raising meat chickens?
*(follow chat and ask probing questions as necessary)*	
Wrap-up	Thanks very much for your input everyone. Is there anything else you would like to add?
Final post	I will be in touch within the next couple days about the final part of the study, the post-forum survey. Thank you again for taking the time to participate in the chat.

**Table 3:** Group composition, number of posts, and number of words per chat.

Chat	Number of Participants *				Number of Posts			Total Number of Words
	Ind	Anim Adv	Gen Pub	Other	Directed towards Moderator	Directed towards Others ^1^	Total	
Chat 1	-	5	-	-	78	40	141	3214
Chat 2	1	3	2	-	62	148	245	5464
Chat 3	2	2	2	1	53	65	160	3684
Chat 4	-	-	2	-	46	3	92	2320
Chat 5	-	1	-	2	68	14	120	2845
Chat 6	-	1	1	-	26	4	49	2002

* Ind = industry-affiliated; Anim Adv = animal advocacy group member; Gen Pub = member of the general public; Other = research or veterinary worker not clearly affiliated with industry or animal advocacy; **^1^** Posts directed towards others did not include posts by the moderator.

**Table 4:** Percentage of participants who correctly answered objective knowledge items on the pre- and post-chat surveys.

Item	Percentage Correct	
	Pre-Chat	Post-Chat
Free range chickens—Chickens that have access to an outdoor area at least during the day as they please	57	62
Beak-trimming—Involves the removal of the tip of the chicken’s beak to prevent feather pecking and cannibalism	95	81
Antibiotics—Are administered to chickens to prevent and treat infections and disease	62	81
Intensive meat chicken farming—Keeping meat chickens indoors at high intensities	43	71
Age of meat chickens at slaughter—5 to 7 weeks	62	76

## Supplementary Materials

The following are available online at www.mdpi.com/2076-2615/6/11/67/s1, Table S1: Chicken welfare composite scores between groups (M: mean, SD: Standard Deviation). Response options ranged from 1 (very low) to 5 (very high).
