# Supplementary material for: Online Chats to Assess Stakeholder Perceptions of Meat Chicken Intensification and Welfare
Source: Animals (Basel). 2016 Oct 27;6(11):67. doi: 10.3390/ani6110067 (PMC5126769; doi:10.3390/ani6110067)
Supplement: Supplementary file 1 [file animals-06-00067-s001.pdf]

Supplementary Materials: Online Chats to Assess Stakeholder Perceptions of Meat Chicken Intensification and Welfare

Tiffani J. Howell, Vanessa I. Rohlf, Grahame J. Coleman and Jean-Loup Rault

Table S1. Chicken welfare composite scores between groups (M: mean, SD: Standard Deviation). Response options ranged from 1 (very low) to 5 (very high).

Item		Chicken Husbandry/Welfare Self-Rated Knowledge	Australian Meat Chicken Practices Self-Rated Knowledge	Natural Living	Protection	General Welfare
**Pre-chat**						
Total	M	3.26	3.39	4.70	4.25	2.12
	SD	0.99	0.92	0.66	0.66	1.10
Industry	M	4.67	4.67	3.44	4.60	4.11
	SD	0.58	0.58	0.77	0.35	1.02
Animal advocacy	M	3.13	3.25	4.88	4.08	1.88
	SD	0.84	0.89	0.35	0.87	0.75
General public	M	2.87	3.00	5.00	4.30	1.63
	SD	0.84	0.58	0.00	0.49	0.45
**Post-chat**						
Total	M	3.68	3.72	4.61	4.42	2.16
	SD	0.82	0.83	0.71	0.75	1.19
Industry	M	5.00	5.00	3.67	4.67	4.11
	SD	0.00	0.00	0.88	0.31	1.02
Animal advocacy	M	3.63	3.50	4.71	4.33	1.96
	SD	0.52	0.76	0.70	1.03	1.00
General public	M	3.25	3.43	4.88	4.43	1.63
	SD	0.71	0.54	0.35	0.57	0.58
Effect of time	λ	**0.77 ***	**0.70 ***	1.00	0.87	0.99
(df = 1,16)	F	**4.69 ***	**6.58 ***	0.07	2.31	0.13
Effect of group (df = 2,16)	F	**9.69 ****	**7.51 ****	**10.09 ****	0.42	**12.52 ****

* Effect is significant at $p < 0.05$; ** Effect is significant at $p < 0.01$.

© 2016 by the authors; licensee MDPI, Basel, Switzerland. This article is an open access article distributed under the terms and conditions of the Creative Commons by Attribution (CC-BY) license (http://creativecommons.org/licenses/by/4.0/).
